# Lung squamous cell carcinoma with solitary ocular metastasis and its successful treatment with thoracic surgery and chemotherapy: an interesting and rare case report

**DOI:** 10.1186/s12885-018-4944-y

**Published:** 2018-10-20

**Authors:** Ye Guo, Xu Wang, Jun Xiao, Yinghui Xu, Yangyang Cai, Chao Sun, Kewei Ma

**Affiliations:** 1 The Jilin University First Hospital, No. 71, Xinmin Street, Changchun, Jilin, 130021 People’s Republic of China; 2 The Jilin University Second Hospital, No. 218, Ziqiang Street, Changchun, Jilin, 130021 People’s Republic of China

**Keywords:** Solitary ocular metastasis, Lung squamous cell carcinoma, Multidisciplinary treatment

## Abstract

**Background:**

The incidence of ocular metastasis from lung cancer is reported to be 0.1–7%, with adenocarcinoma and small cell lung cancer accounting for the highest proportions of these cases. The majority of cases involves metastasis to more than one other distal organ in addition to the eye. Here, we report for the first time, a case of lung squamous cell carcinoma with solitary symptomatic ocular metastasis as the initial manifestation that was managed by a multidisciplinary treatment (MDT).

**Case presentation:**

A woman presented at the ophthalmology department of hospital with a 1-week history of left eye pain and blurred vision. Systemic examination led to the diagnosis of central lung cancer in the right lower lobe with ocular metastasis. After consultations with an MDT, including specialists from the surgery, internal medicine, ophthalmology, radiotherapy and imaging departments, the patient underwent surgery and chemotherapy. Her eye symptoms disappeared, and the ocular lesion was well controlled without any specific ocular treatment. The patient demonstrated a prolonged progression-free survival.

**Conclusion:**

This is the first report of a rare case with solitary ocular metastasis as the initial manifestation of lung squamous cell carcinoma. This rare patient was treated based on evidence-based medicine, indicating the importance of cooperation within an MDT. The successful treatment of this case was reported as a new therapeutic reference for clinicians who encounter similar cases in the future.

## Background

The incidence of ocular metastases from lung cancer is reported to be 0.1–7% in the global literature [1–3], with adenocarcinoma and small cell lung cancer accounting for the highest proportions of these cases [1, 2]. The majority of cases involve metastasis to more than one other distal organ in addition to the eye [4, 5]. However, lung squamous cell carcinoma with solitary symptomatic ocular metastasis as the initial manifestation that is managed by a multidisciplinary treatment (MDT) has never been reported before.

## Case presentation

**Fig. 1:**
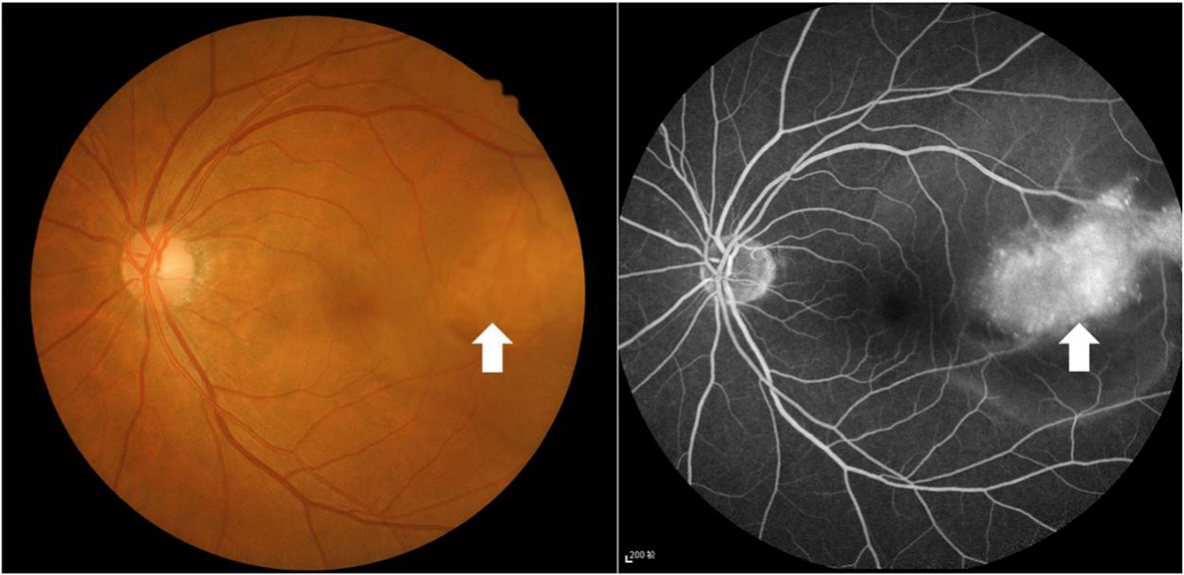
Ophthalmic fundoscopy shows the temporal side of the left eye with a yellow and white macular mass of approximately 8 PD, visible peripheral choroidal folds, and retinal detachment involving the macula and the fovea

**Fig. 2:**
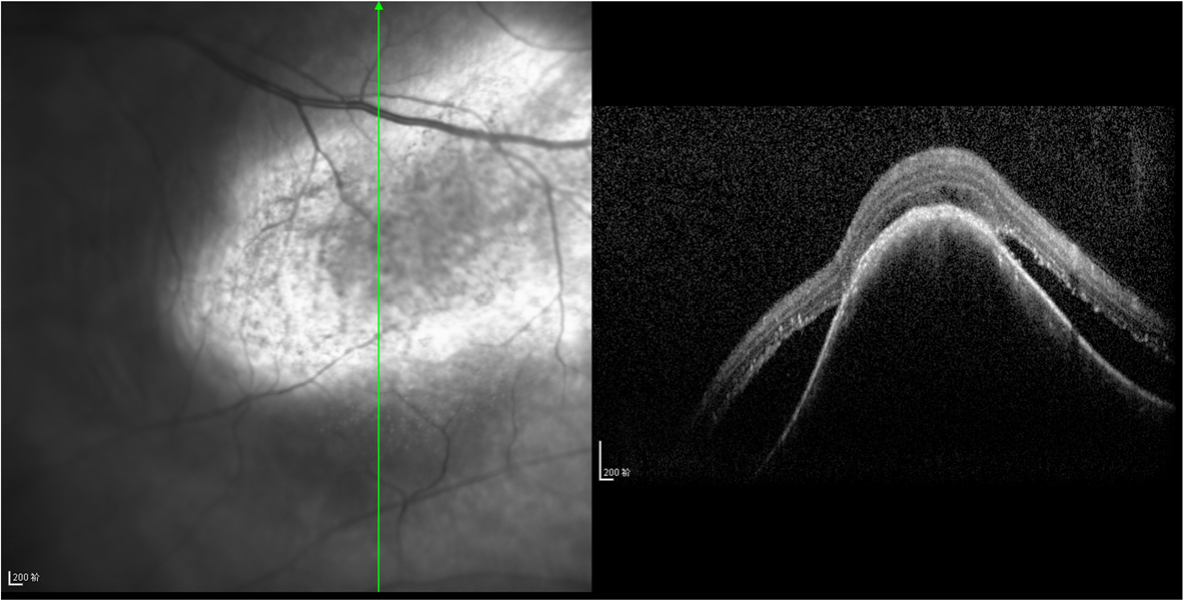
OCT: visible lesions in the choroidal uplift and retinal edema

**Fig. 3:**
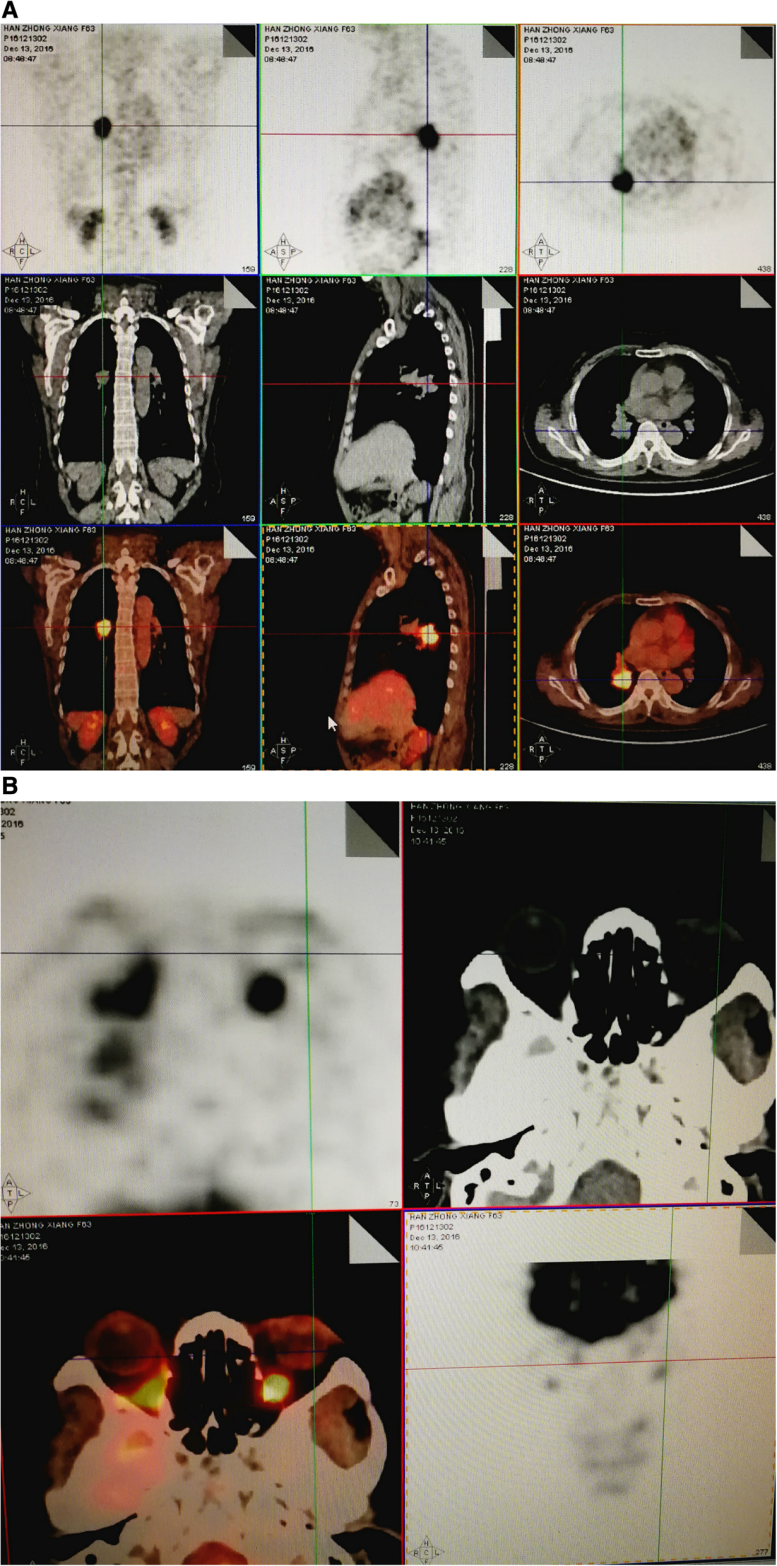
**a** PET-CT showing right lower lobe central lung cancer. **b** Localized thickening of the left fundus; early and delayed metabolic signals are not high, and other tests for tuberculosis were proposed

**Fig. 4:**
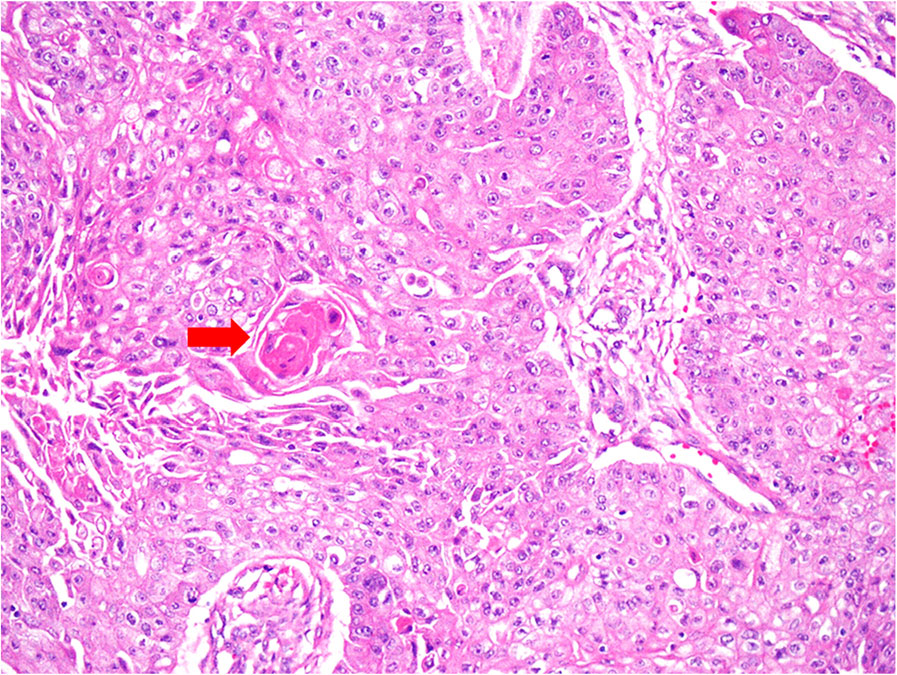
The figure shows the pathology after bronchoscopic biopsy. Microscopically, the tumor tissues are arranged in sheets and nests. Some neoplastic cell-nests have peripheral palisading, with typical keratinized peals (→) in the center. Tumor cells are light to moderate heteromorphic, with intercellular bridges. (H&E staining 200 times magnification). The diagnosis is lung squamous cell carcinoma

**Fig. 5:**
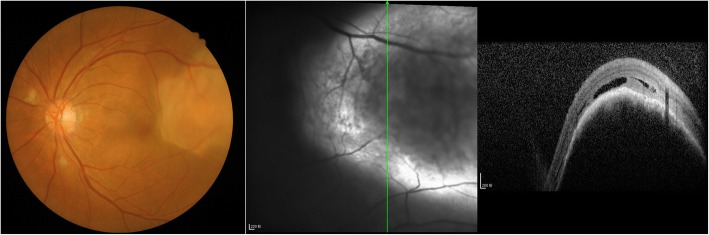
Eye examination after thoracic surgery shows a significantly larger mass than before

**Fig. 6:**
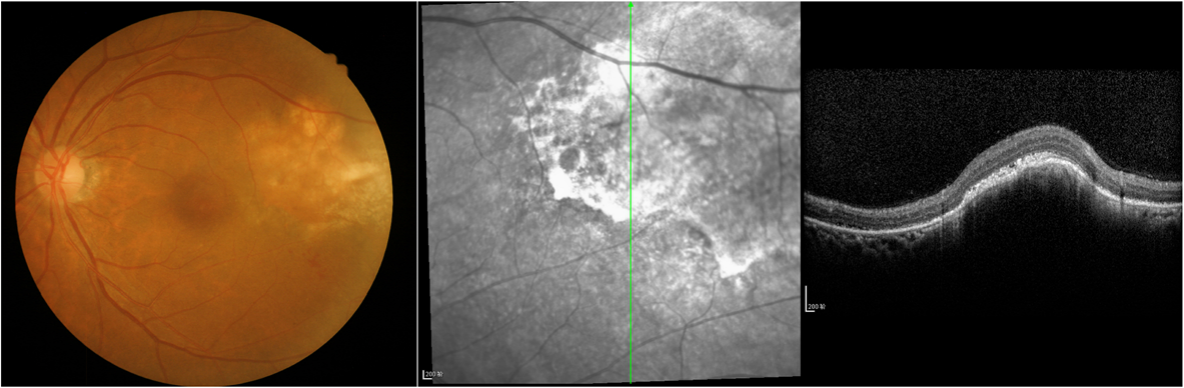
Eye examination after 2 courses of chemotherapy shows significant reduction in the mass. Ophthalmologists decided that the chemotherapy was effective for ocular lesions

**Fig. 7:**
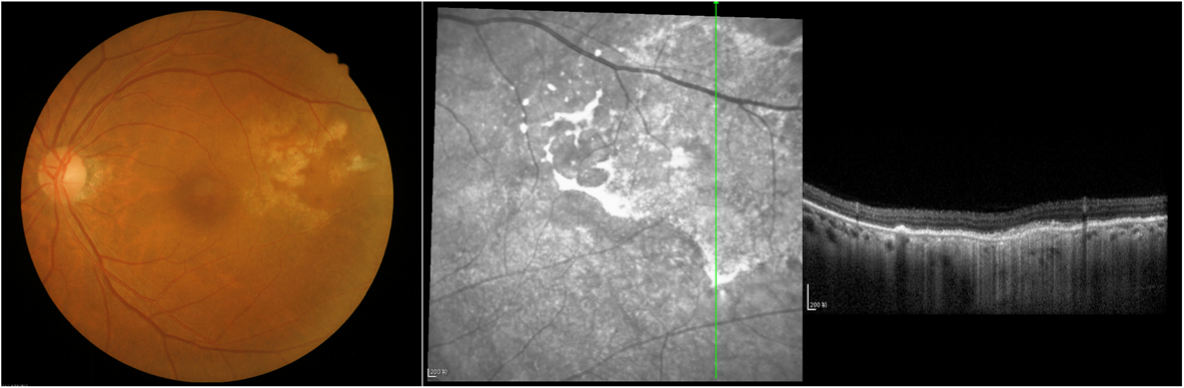
Eye examination by imaging after 4 courses of chemotherapy shows complete disappearance of the tumor. The residual lesion indicates the presence of retinal epithelial pigment disorder

A 62-year-old woman presented at the ophthalmology department of hospital with a 1-week history of left eye pain and blurred vision. The ophthalmologist performed ophthalmic fundoscopy and optical coherence tomography (OCT) on the patient (Fig. 1 and Fig. 2). The diagnosis was metastatic carcinoma in the eye. The diagnostic evaluation was completed with PET-CT, which confirmed central lung cancer in the lower lobe of the right lung with ocular metastasis (Fig. 3). Brain MRI showed no obvious abnormality. Pathological findings of bronchoscopic biopsy indicated non-small cell lung cancer (NSCLC) in the lower segment of the right lower lobe of the right lung with features of squamous cell carcinoma (Fig. 4). After consultations with an MDT, including specialists from the surgery, internal medicine, ophthalmology, radiotherapy and imaging departments, the patient underwent right lower lobe resection and lymph node dissection in December 2016. Postoperative pathology led to the diagnosis of right lung squamous cell carcinoma of T2aN1 stage. In February 2017, the patient underwent an eye examination, which indicated that the ocular lesions were enlarged (Fig. 5). The patient received 4 courses of gemcitabine plus cisplatin regimen from February to May 2017. Her eye symptoms improved after 2 courses of chemotherapy (Fig. 6) and disappeared completely after 4 courses (Fig. 7). The progression-free survival (PFS) duration of the patient was 11.9 months until the emergence of brain metastasis, which was treated with cranial radiotherapy. The patient was followed up for 16.5 months (March 2018) after surgery, and the ocular lesion was still well controlled without any specific ocular treatment.

## Discussion

Clinical reports of intraocular metastasis are rare and often confined to single cases, and only a few studies (with small sample sizes) have evaluated this condition. The majority of cases are accompanied by metastasis to other major organs such as the brain, liver, kidney, and adrenal glands [6]. To the best of our knowledge, there are only 5 case reports of lung squamous cell carcinoma with intraocular metastasis thus far [7–11], Table 1). Klein et al. [7] reported on a male patient with previously documented chronic lymphocytic leukemia who suffered from poorly differentiated lung squamous cell carcinoma with retinal metastasis. It was a very simple case with descriptions of the clinical features of ocular metastasis without information on the TNM stage, treatment or prognosis of lung cancer. Asteriou et al. [8] described a locally advanced case accompanied by other metastases including choroidal metastasis. Although chemotherapy and radiotherapy were performed, their effect or disease prognosis was not recorded. Yalcinbayir et al. [9] described a lung squamous cell carcinoma case with brain and retinal metastases. In that case, the interval from the diagnosis of primary cancer to the diagnosis of retinal metastasis was 18 months. The patient was treated with chemotherapy and cranial radiotherapy, and the retinal lesion displayed marked regression without other specific ocular treatment. The patient is at the ninth month of follow-up, and the retinal lesion is still well controlled. Hiraki et al. [10] described a cT2N2M1 lung squamous cell carcinoma case with brain metastasis in which the symptoms of iris metastasis appeared during the second course of chemotherapy. Radiotherapy was administered to the left eye and brain, and the overall survival (OS) of the patient was approximately 6 months. Prathipati et al. [11] reported the occurrence of lung squamous cell carcinoma in a 35-year-old female with choroidal metastasis as the initial presentation, who had undergone treatment for hypopharynx carcinoma as metachronous second primary. Postradiotherapy follow-up indicated a significant decrease in left eye pain and stabilization of vision. Although chemotherapy was planned, there was no record of the effect or prognosis (Table 1). Here, for the first time, we document a stage T2aN1M1 lung squamous cell carcinoma with solitary symptomatic ocular metastasis.

**Table 1:** Five case reports of lung squamous cell carcinoma with intraocular metastasis

Age/s Sex	Lung cancer location	Ocular involvement	Tumor location	Symptom	Ocular symptoms are the initial symptoms	Other metastatic sites	Systemic treatment/Response	Ocular treatment/Response	OS (months)	First author/Year	Thoracic operation	managed by MDT
52/M	Left upper lobe	Retina	LE&RE	Decreased visual acuity	Y	Brain, meninges, lymph nodes, liver, adrenal glands, spleen, kidney, lungs, heart	NA	NA	2	Ronald Klein/1977	N	N
72/F	Right lower lobe	Iris	LE	Ocular pain, blurred vision	N	Brain	Chemo (vindesine, cisplatin)/PR	Radio /CR	6.3	Akio Hiraki/1998	N	N
46/M	Right upper lobe	Choroid	LE	Reduction in vision with concomitant blurriness	Y	Adrenal, brain, bone	Chemo/NA	Radio/NA	NA	Christos Asteriou/2010	N	N
35/F	Right upper lobe	Choroid	LE	Pain, blurred vision	Y	None	Chemo(paclitaxel, cisplatin)/NA	Radio/SD	NA	Archana Prathipati/2016	N	N
54/M	NA	Retina	LE	Blurred vision	N	Brain	Chemo(paclitaxel, carboplatin)/PR; Chemo(docetaxel, carboplatin )/PR	None	NA (follow-up 9 months)	Ozgur Yalcinbayir/2017	N	N

Intraocular metastasis is commonly located in the posterior uvea but rarely in the iris or ciliary body. Choroidal metastasis is the most frequent intraocular lesion [12]. The higher incidence of choroidal metastasis than of other ocular lesions may be because choroidal lesions are more symptomatic and hence easier to diagnose [13, 14]. In addition, the abundant supply of posterior ciliary arteries to the choroid may be conducive to metastasis [15]. The incidence of metastasis to the left eye is higher than that to the right eye because the left common carotid artery branches directly from the aortic arch, while the right common carotid artery branches from the brachiocephalic trunk, so that cancer cells reach the left eye more directly than they reach the right eye [13]. The intraocular metastatic lesions in our patient were located in the choroid of the left eye, in line with previous studies [12, 13].

Metastasis is considered to occur at the final stages of the disease, by which time the majority of patients already experience typical lung cancer symptoms. A decrease in vision and blurred vision due to choroidal metastasis as the initial manifestation are therefore very rare. In the present study, we described a patient with lung squamous cell carcinoma that was initially diagnosed from choroidal metastasis, indicating that non-ocular primary tumors should always be considered probable causes of choroidal masses. In particular, ophthalmologists should be aware of this rare condition, and detailed systemic evaluation should be performed.

Currently, a multidisciplinary approach is very important in the diagnosis and treatment of lung cancer, especially for rare cases. Treatment is often controversial in the case of uncommon metastases including solitary ocular metastasis. These patients should be managed using evidence-based medicine involving the cooperation among ophthalmologists, surgeons, oncologists, and radiologists, along with others. We describe the following treatment strategies: (i) Systemic therapy: Traditionally, systemic therapy, such as chemotherapy or target therapy, is recommended as the standard therapy for metastatic disease. However, the effect of treatment on ocular metastatic lesions has not been widely reported. A: Some case reports show that chemotherapy is efficient for choroidal metastasis from NSCLC [5, 9, 16–20], mostly related to adenocarcinoma [5, 16–20]. Yalcinbayir et al. [9] described a lung squamous cell carcinoma patient who received docetaxel/carboplatin combination chemotherapy, and the retinal metastatic lesion displayed marked regression without other specific ocular treatment. Several reports have shown that systemic chemotherapy alone using gemcitabine-platinum [5, 18] or pemetrexed-cisplatin [19] for lung adenocarcinoma can lead to complete involution of the choroidal metastasis with improvements in visual acuity. Studies from George et al. [17] and Makabe et al. [16] suggested that systemic chemotherapy together with bevacizumab may have a role in improving vision and survival of lung adenocarcinoma patients. However, bevacizumab is not recommended for lung squamous cell carcinoma patients. B: The effectiveness of targeted therapy alone and of small molecule receptor tyrosine kinase inhibitors (TKIs) alone, such as gefitinib, erlotinib, crizotinib and alectinib, has been reported in the literature for NSCLC patients with choroidal metastasis [21–23], but these treatments are mainly suitable for lung adenocarcinoma, as the incidence of the corresponding driver mutations is too low in lung squamous cell cancer. (ii) Surgery: Albain et al. [24] analyzed 2531 patients with extensive-stage NSCLC and found that patients with isolated metastatic lesions had better prognosis after surgical resection than after chemotherapy alone, radiotherapy alone, or best supportive care. The NCCN guidelines and Chinese consensus [25] recommend local (surgery or radiotherapy) plus systemic treatment (chemotherapy or targeted therapy) for primary NSCLC lesions of stage of T1-3 N1 with solitary metastasis. However, there is no mention of the treatment for ocular metastasis in the guidelines because of the very low incidence of this condition. (iii) Localized ocular therapy: The aim of localized ocular treatment is to restore visual acuity and therefore improve the patients’ quality of life for their remaining life span. Available regimens with external beam radiation, plaque radiotherapy, laser photocoagulation, intravitreal bevacizumab and surgical resection have been proposed to treat choroidal metastasis [26–28]. Nevertheless, complications, such as cataract, retinopathy, glaucoma, or even blindness, should be noted when patients undergo radiotherapy. The prognosis of the patients with intraocular metastasis depends on the overall therapeutic effect on lung cancer. However, these patients are rarely included in prognostic analyses. Wiegel et al. [29] revealed an average survival of 7 months following a diagnosis of uveal metastases. A retrospective study from China analyzed 21 patients with lung cancer with ocular metastasis, and 15 patients underwent systemic chemotherapy. The median OS was 12 months, and the 1-year survival rate was 44.7% [29]. Yan et al. [30] reviewed relevant literature and analyzed 30 patients with survival information. The median OS was 13.00 months, but for patients who underwent systemic chemotherapy alone, the OS was only 10 months. The presence of ocular metastasis usually indicates a poor outcome. However, certain patients with oligometastases may benefit from systemic treatment plus local treatment, with a median OS of 13.5 months [31].

Considering the clinical condition of the patient, thoracic surgery was the first treatment decided at the MDT meeting according to the efficiency and prognostic data described above. Pathological assessment confirmed the diagnosis of squamous cell carcinoma, and thus, neither bevacizumab nor TKIs were a better choice than chemotherapy. Thus, the patient was subjected to chemotherapy after the thoracic surgery, and radiotherapy was recommended for the metastatic lesions if deemed necessary. The treatment was very effective. The choroidal metastatic lesions displayed marked regression. A study by Shah et al. [32], spanning four decades, suggested that combination treatment is superior to systemic treatment alone in the overall and ocular management of choroidal metastasis from lung cancer. In our case, the patient was managed by an MDT after chemotherapy. Radiotherapists and ophthalmologists believed that the patient responded well to chemotherapy. Local treatments such as external beam radiation, plaque radiotherapy, laser photocoagulation and surgical resection are accompanied by complication risks. Klaus et al. [6] reported complications including total exudative retinal detachment, vitreous hemorrhage and blindness. These complications can affect the quality of life of the patient. Thus, close observation is recommended. To date (March 2018), the patient has been followed up for 16.5 months, and her PFS was 11.9 months, longer than previously reported data (30).

## Conclusion

In summary, this is the first report of a rare case of solitary ocular metastasis as the initial manifestation of lung squamous cell carcinoma. This rare patient was treated with evidence-based medicine, which indicated the importance of cooperation within an MDT. To date (March 2018), the patient has been followed up for 16.5 months, and her PFS was 11.9 months. Her visual acuity improved after chemotherapy. The successful treatment of this case was reported as a new therapeutic reference for clinicians who encounter similar cases in the future.

## Abbreviations

MDT: Multidisciplinary treatment
NSCLC: Non-small cell lung cancer
OCT: Optical coherence tomography
PD: Papilla diameter
PFS: Progression-free survival
